# Alcohol and drug use as factors for high-school learners’ absenteeism in the Western Cape

**DOI:** 10.4102/sajpsychiatry.v27i0.1679

**Published:** 2021-12-14

**Authors:** Godswill N. Osuafor

**Affiliations:** 1 Population Studies and Demography, North-West University, Mafikeng, South Africa

**Keywords:** school absenteeism, alcohol use, cannabis smoking, learners, longitudinal study

## Abstract

**Background:**

School absenteeism has been studied in detail in relation to health risk behaviours using cross sectional studies.

**Aim:**

The aim of this longitudinal study was to examine the association amongst alcohol, drug use and high-school learners’ absenteeism.

**Setting:**

This study was set in the Western Cape.

**Methods:**

Data were collected at three separate time points from 2950, 2675 and 2230 grade 8 learners aged 13–18 years old on school absenteeism, alcohol and drug use and sociodemographic characteristics. Associations between school absenteeism, alcohol and cannabis and sociodemographic factors use were examined using descriptive and chi-square analyses. Binary logistic regression was performed using generalised linear mixed model analyses.

**Results:**

Results revealed that 9.3% of the learners were absent for 2 weeks in the 15 weeks of the school year. Alcohol consumption (*X*^2^ = 34.1, *p* < 0.001; odds ratio [OR]: 1.64 (1.38–1.94), *p* < 0.001) and smoking cannabis (*X*^2^ = 49.9, *p* < 0.001; OR: 2.01 (1.65–2.45), *p* < 0.001) were associated with school absenteeism at bivariate and multivariate analyses. Furthermore, alcohol (OR: 1.42 (1.06–1.89), *p* < 0.05) and cannabis (OR: 1.57 (1.11–2.22), *p* < 0.05) use remained robust in predicting learners school absenteeism after adjusting for age, sex and socioeconomic status.

**Conclusion:**

These findings suggest that alcohol consumption and smoking cannabis are contemporary factors associated with school absenteeism. Therefore, interventions to ensure learners’ consistent attendance to school should integrate prevention of alcohol and cannabis use.

## Introduction

Substance use has been studied in great detail in relation to adolescents’ health risk behaviours in South Africa. However, studies on substance use and school absenteeism have not been adequately investigated. Associations between substance use and absenteeism were conspicuously missing in several studies conducted among school going adolescents in South African provinces. In Limpopo province, studies have shown that alcohol use among school going adolescents was a serious public health concern.^1,2,3^ In another study in the Western Cape province, alcohol use and other drug use were associated with risky sexual behaviours between grades 8–10 learners.^4^ Govender et al.^5^ in multi-system model of risk and protective factors found that alcohol consumption by adolescents was associated with sexual risk behaviour in KwaZulu-Natal (KZN) province. Of all these studies conducted among school going adolescents in South Africa, none investigated the relationship between substance use and school absenteeism.

Studies elsewhere have also neglected the association between substance use and adolescent school absenteeism^6^ in a cross-sectional study that examined school absenteeism amongst 704 students aged 10–15 years in Delhi. They found that age, male sex, increasing birth order, low parental education and income predicted school absenteeism. The study further revealed that school truancy, school phobia and family reasons were predictors of school absenteeism. Despite the robustness of the study, the link between substance use and school absenteeism was not investigated.

There is paucity of literature that overtly assesses the nexus of substance use and school absenteeism. In a cohort of 1259 and 1076 grade 9 learners, alcohol consumption was associated with poor academic performance and absenteeism in KZN, South Africa.^7^ Heradstveit et al.^8^ studied the association amongst alcohol, drug use and a low-grade point average (GPA) and school attendance in Norway. They utilised registry-based data on school-related functioning from 7874 adolescents and found that alcohol and drug consumption were consistently associated with low GPA and school absenteeism. The study further demonstrated that alcohol and illicit drug use remained statistically significant predictors of school-related negative outcomes after adjusting for gender, age, socioeconomic status (SES) and mental health problems. The authors concluded that alcohol and drug use are important factors for school-related problems independent of mental health problems in Norway. Soares et al.,^9^ in a cross-sectional study, examined the association between the consumption of alcohol and other drugs with school absenteeism and found that high-school children who consumed alcohol, tobacco, inhalant products and marijuana were prone to be absent from school in Brazil.

School absenteeism is an indicator of social exclusion and is a risk factor for selfharm and suicidal ideation in children and adolescents.^10^ Aside from negative outcomes of school absenteeism, few studies have demonstrated that consumption of alcohol and drugs have several consequences ranging from school absenteeism,^9,11^ school disengagement,^11^ poor academic performance^6,12^ and ultimately dropping out of school.^13,14,15^ A hierarchical longitudinal study of grade 9–12 leaners showed that frequent binge drinking was associated with low likelihood of high academic performance and school engagement in Ontario and Alberta, Canada.^12^ Other studies in South Korea^16^ and France^17^ have shown association between illicit drug use and other school negative consequences.

School absenteeism is a crucial matter with a multitude of negative consequences. Furthermore, the relationship between chronic absenteeism and substance use is difficult to fully understand because of methodological issues. Gakh et al.^18^ in an integrative literature review demonstrated that the relationship between school absenteeism and substance use provided a limited understanding of how and why this association manifests. Using cross-sectional and local-level data often limits nuanced investigation on substance use and school absenteeism. Therefore, Gakh et al.^18^ called for absenteeism research that uses longitudinal methods and national data, which articulate methodologies and self-appraised limitations. There is a deficit of longitudinal studies on substance use and school absenteeism in South Africa. Therefore, the present study was designed to examine association between substance use and adolescents school absenteeism using longitudinal data.

## Methods

### The study method

The Western Cape Department of Health, the Western Cape Department of Basic Education and a number of non-governmental organisations (NGOs) in conjunction with the Centre for the Support of Peer Education rolled out a longitudinal survey on the Evaluation of Peer Education in Western Cape Schools Programme (EPEP). Data were collected on change in attitudes, knowledge and behaviour on a set of pre-identified indicators in relation to peer education and human immunodeficiency virus/acquired immune deficiency syndrome (HIV/AIDS) from grade 8 and grade 9 learners between February 2012 and June 2013. The present study was a longitudinal quantitative secondary research derived from the EPEP data.

### The study participants

Grade 8 learners were recruited from 236 schools in the Western Cape. Data were collected at three-point times from 2950, 2675 and 2230 learners of age range 13–18 years. Data were collected from the respective schools using a self-administered survey questionnaire accessible in English, Afrikaans and Xhosa through the assistance of trained fieldworkers.

### Measured variables

The dependent variable examined in the study was school absenteeism. We used Kearney’s definition of problematic absence of having more than 2 weeks (15%) absenteeism during the 15 weeks in the school year.^19^ Learners who reported absence from school for 2 weeks were coded 1; otherwise they were coded 0.

The main independent variables were alcohol and cannabis use. To measure substance use, learners were asked whether they had consumed alcohol in the past 6 months prior to the survey. They were further asked if they have consumed cannabis in the past 6 months before the survey. Those who reported ‘yes’ to having consumed alcohol or smoked cannabis were assigned 1; otherwise they were assigned 0 suggesting no use of alcohol or cannabis.

Other independent variables included were learners’ socio-demographic characteristics (age, sex, population group, place of residence, family structure, SES). Learners were asked to respond to four^4^ statements on basic human needs: first, ‘we don’t have enough money for food’; second, ‘we have enough money for food, but not other basic items such as clothes’; third, ‘we have enough money for food and clothes, but are short for other things’ and; fourth, ‘we have enough money for food and clothes, and also a bit extra for other things’. Responses 1 and 2 were categorised as low SES; responses 3 and 4 were categorised as middle and high SES, respectively. School factors were assessed on participation in a peer education programme and participation in school sports. Finally, individual factors were assessed on participation in religious activities and attending a youth group for HIV/AIDS.

### Data analysis

The three-time point data were restructured, and generalised linear mixed model analyses were performed using Statistical Package for the Social Sciences (SPSS) version 25. Given the relevance of sex, substance use and school absenteeism in the study, non-responses to sex, substance use and school absenteeism were excluded from the analysis. Descriptive statistics was carried out for all the variables at the univariate level. Chi-square analyses were carried out to ascertain the association between the predictor variables and school absenteeism. Predictor variables that were not significant at bivariate level were removed at multivariate analyses. At multivariate analyses, unadjusted and adjusted models of predictors of school absenteeism were presented as odd ratios (OR) and 95% confidence interval (CI).

### Ethical considerations

Ethics approval was obtained from the Western Cape Education Department and Research Ethics Committee of the Human Sciences Research Council (HSRC), which is nationally accredited by the South African Government. Given that most learners were under the age of 18 years, approval of the parent or guardian was sought by completing the informed consent forms. Furthermore, assent was obtained from the learners who participated in the study.

## Result

Table 1 showed that 1 in 10 of the learners had been absent from school for 2 weeks during the 15 weeks in the school year. Two-third of the learners were 14 years old. Over a quarter (34.4%) have consumed alcohol, whereas less than a quarter (16.0) have smoked cannabis in the past 6 months. Black and coloured learners constituted 48.3% and 47.7% of the population group, respectively. Slightly over half (55.5%) of the learners were females. Half (50%) of the learners were residing in the urban area. About two-fifth of the learners belong to high-SES status. In terms of family structures, about half were living with single parents and over a quarter with both parents. About two-third (61.5%) participated in school sports, whereas less than a quarter (16.0%) participated in peer education programmes. With regard to individual factors, the majority (78.1%) of the learners participated in religious activities, and about two-third had never participated in youth group for HIV and AIDS in the last 12 months.

**TABLE 1:** Sociodemographic profile of the learners in selected schools in the Western Cape.

Variables	Number of observations	Percent
**Absent from school for 2 weeks**		
No	6211	90.7
Yes	636	9.3
**Age (years)**		
≤ 13	2397	30.5
14	2398	30.5
15	1372	17.5
16+	1688	21.5
**Consumed alcohol in the last 6 months**		
No	4962	65.6
Yes	2606	34.4
**Smoked cannabis in the last 6 months**		
No	6315	84.0
Yes	1202	16.0
**Population group**		
Black	3592	48.3
Coloured	3546	47.7
White	300	4.0
**Sex**		
Male	3429	44.5
Female	4281	55.5
**Place of residence**		
Rural	1450	19.7
Sub-urban	2222	30.3
Urban	3673	50.0
**Socioeconomic status**		
Low	1792	24.4
Middle	2207	30.1
High	3335	45.5
**Family structure – care giver**		
Single parents	3365	50.9
Both parents	2018	30.5
Others	1231	18.6
**Participate in sport – at school**		
Never	2716	38.5
Always	4346	61.5
**Participated in a peer education programme?**		
No	4745	64.7
Participant	1414	19.3
Educator	1172	16.0
**Go to church or choir or youth group or Muslim school**		
Never	1583	21.9
Always	5643	78.1
**Attended a youth group for HIV and AIDS in the last 12 months**		
No	3045	65.8
Yes	1583	34.2

HIV, human immunodeficiency virus; AIDS, acquired immune deficiency syndrome.

Table 2 presents the associations amongst school absenteeism, substance use and socio-demographic factors. Using alcohol (*p* < 0.001) and smoking cannabis (*p* < 0.001) during the last 6 months was significantly related to the 2 weeks of school absenteeism.

**TABLE 2:** School absenteeism relationship with substance use and sociodemographic factors.

Variables	Observations	No		Yes		*X^2^*	*P*-value
	*N*	%	*N*	%			
**Consumed alcohol in the last 6 months**						**34.110**	**0.000**
No	4445	4101	92.3	344	7.7		
Yes	2246	1974	87.9	272	5.1		
**Smoked cannabis in the last 6 months**							
No	5631	5173	91.9	458	8.1		
Yes	1013	860	84.9	153	15.1		
**Age (years)**						**87.579**	**0.000**
≤ 13	2172	2036	93.7	136	6.3		
14	2153	1990	92.4	163	7.6		
15	1165	1028	88.2	137	11.8		
16+	1356	1156	85.3	200	14.7		
**Population group**						** 11.253**	**0.004**
Black	3145	2822	89.7	323	10.3		
White	257	232	90.3	25	9.7		
Coloured	3157	2909	92.1	248	7.9		
**Sex**						**18.015**	**0.000**
Male	2988	2660	89.0	328	11.0		
Female	3804	3501	92.0	303	8.0		
**Place of residence**						**11.828**	**0.003**
Rural	1243	1106	89.0	137	11.0		
Sub-urban	1950	1750	89.7	200	10.3		
Urban	3286	3019	92.0	267	8.1		
**SES**						**57.987**	**0.000**
Low-SES	1532	1322	86.3	210	13.7		
Middle-SES	1944	1761	90.6	183	9.4		
High-SES	3013	2808	93.2	205	6.8		
**Family structure – care giver**						**17.027**	**0.000**
Single parents	2968	2674	90.1	294	9.9		
Both parents	1809	1682	93.0	127	7.0		
Others	1063	944	88.8	119	11.2		
**Participate in sport – at school**						**1.965**	**0.161**
Never	2393	2197	91.8	196	8.2		
Always	3870	3513	90.8	357	9.2		
**Participated in a peer education programme?**						**13.244**	**0.001**
No	4199	3844	91.5	355	8.5		
Participant	1279	1134	88.7	145	11.3		
Educator	1033	919	89.0	114	11.0		
**Participated in religious activities**						**2.929**	**0.087**
Never	1377	1238	89.9	139	10.1		
Always	5029	4596	91.4	433	8.6		
**Attended a youth group for HIV and AIDS in the last 12 months**						**2.491**	**0.114**
No	2723	2489	91.4	234	8.6		
Yes	1377	1238	89.9	139	10.1		

HIV, human immunodeficiency virus; AIDS, acquired immune deficiency syndrome; SES, socioeconomic status.

Significant at ***, *p* < 0.000; **, *p* < 0.01, and *, *p* < 0.05.

Sociodemographic risk factors associated with higher percentages of school absenteeism included: increasing age, low SES, being black, being male, residing in rural area and living with grandparents. School factors showed that participating in school sports was not associated with absenteeism from school. Surprisingly, participating in a peer education programme as educator showed highest percentage in school absenteeism. However, participating in religious activities and attending a youth group for HIV and AIDS was not associated with school absenteeism.

Table 3 presents the results from the multivariable models. In both unadjusted and adjusted analyses, alcohol consumption and smoking cannabis significantly increased the likelihood of school absenteeism amongst learners. Similarly, increasing in age significantly predicted school absenteeism. Compared to learners from low-SES, those from middle-SES and high-SES showed 43% and 42% reduced odds of school absenteeism. Similarly, learners living with both parents had 40% reduced odd of school absenteeism compared with those residing with single parents. Again, learners were 1.5 times more likely to be absent from school if they were participants in a peer education programme compared to those who did not participate in the programme. However, in unadjusted models, sex, population group and place of residence were significantly associated with school absenteeism that disappeared in the adjusted analyses.

**TABLE 3:** Predictive models of school absenteeism based on multivariate regression analyses.

Variables	Unadjusted odds ratio	95% CI	Adjusted odds ratio	95% CI
**Consumed alcohol in the last 6 months**				
No (ref)	1.000	-	1.000	-
Yes	1.643***	1.389–1.943	1.417*	1.061–1.891
**Smoked cannabis in the last 6 months**				
No (ref)	1.000	-	1.000	-
Yes	2.009***	1.651–2.446	1.571*	1.112–2.217
Age	1.398***	1.300–1.504	1.197**	1.062–1.349
**Sex**				
Male (ref)	1.000	-	1.000	-
Female	0.702***	0.596–0.827	0.882	0.682–1.140
**Population group**				
Black (ref)	1.000	-	1.000	-
White	0.941	0.613–1.445	1.008	0.756–1.345
Coloured	0.745**	0.626–0.886	0.930	0.449–1.929
**Place of residence**				
Rural (ref)	1.000	-	1.000	-
Sub-urban	0.923	0.733–1.161	1.066	0.751–1.513
Urban	0.714**	0.575–0.887	1.276	0.876–1.858
**SES**				
Low-SES (ref)	1.000	-	1.000	-
Middle-SES	0.654***	0.530–0.808	0.574**	0.412–0.799
High-SES	0.460***	0.375–0.563	0.579**	0.415–0.808
**Family structure – care giver**				
Single parents (ref)	1.000	-	1.000	-
Both parents	0.687**	0.553–0.853	0.599**	0.433–0.829
Others	1.147	0.915–1.437	0.975	0.707–1.347
**Participated in a peer education programme?**				
No (ref)	1.000	-	1.000	-
Participant	1.385**	1.129–1.698	1.527**	1.133–2.056
Educator	1.343**	1.075–1.679	1.114	0.778–1.596

CI, confidence interval; ref, reference category; SES, socioeconomic status.

Significant at

*** , *p* < 0.000;

** , *p* < 0.01 and

* , *p* < 0.05.

## Discussion

The aim of this study was to examine the association amongst learners’ alcohol use, smoking cannabis and school absenteeism. Incidence of school absenteeism is escalating with cascades of negative consequences and is thus considered as a public health issue in lower-middle income and high-income countries. Our findings are comparable with other studies,^6,20^ which have documented 7.3% – 17.8% prevalence of absenteeism amongst high school learners.

Other studies have reported 36.2% prevalence when absenteeism was measured in days.^9^

The concern does not end with school absenteeism. However, school absenteeism gives rise to more complex school problems leading to school dropout^13,17,21,22^ and endangered transition to promising adulthood.^13^ Flisher et al.^21^ suggested that preventing episodes of absenteeism has collateral benefits in terms of reducing the extent of dropout as well as poverty alleviation.

The findings corroborate with previous reports that alcohol consumption and smoking cannabis are important factors in adolescents school absenteeism.^9,22,23^ In our study, the association of alcohol and cannabis use with school absenteeism remained statistically significant and robust after adjusting for age, sex, population group and SES. A finding that is consistent with studies in Norway.^8^ However, our findings contradict the studies in Australia and the United States of America that indicated that association between alcohol use and school attendance disappeared when adjusted for confounding factors.^23^ The discrepancies between the findings of the previous studies and the present study could be attributed to the conceptualisation of the confounding variables. Our findings suggest that alcohol consumption and smoking cannabis has exclusive contribution to learners’ school absenteeism.

Few studies have investigated the nexus of alcohol and cannabis use with learners’ school absenteeism along with other confounding factors such as age, sex, population group and socioeconomic factors. We explored the sociodemographic factors contributing to school absenteeism. The finding that increasing age was associated with school absenteeism is consistent with previous reports in New York.^20^ Increasing age may lead to less school connectedness because of alienation with the younger learners. However, this contrasts with studies in Norway^6^ and Tamil Nadu,^24^ which indicated that age was not a factor in school absenteeism.

Consistent with previous findings in Norway, socially economic advantaged learners were at lower risk of being absent from school.^6^ Previous studies in South Korea^16^ and France^17^ have linked school absenteeism with poverty and socially disadvantaged background of the learners. Ingul et al.^25^ further showed that parental unemployment has been associated with adolescents’ school absenteeism.

Our results revealed that learners who participated in peer education programmes were more likely to be absent from school. This is a significant cause for a concern as the purpose of a school peer education programme is to empower the learners for development and self-efficacy against health risk behaviour. The absence of learners from school would mean defeating the aim of school peer education programme that may have far-reaching negative consequences for the learners.

## Limitations

The definition of absenteeism (more than 10 days or 15%) chosen in the study accounted for all aspects of non-attendance with no causal inference.^19^ The study was conducted in the Western Cape province and may fall short in accounting for school absenteeism in other setting within South Africa especially if their substance use patterns and sociodemographic characteristics varies. The present study used longitudinal data to address socioeconomic context of school absenteeism, making it a comprehensive study to assess the nexus of substance use and school absenteeism.

## Conclusion

The study has demonstrated that consumption of alcohol and smoking cannabis are important factors contributing to school absenteeism. Learners who were participants in peer education programmes showed high tendency of absenteeism from school. It seems that their absenteeism may be attributed to not having a specific role to carry out in the school peer education programme. It was unexpected that sex, population group and place of residence did not act as risk factors for absenteeism at adjusted level, given that they showed significant associations at bivariate and unadjusted analyses. However, the highlight of the study was that risk factors for school absenteeism encompassing sociodemographic, family and individual factors were examined in the study.

## Recommendation

Given that alcohol and cannabis use remained robust in predicting school absenteeism, every strategy in reducing episodes of absenteeism should focus on preventing adolescents’ alcohol and cannabis use. Measures should be ensured to identify leaners at risk of alcohol and cannabis use as well as those in low-SES families, as they were prone to be absent from school. Furthermore, school peer education programme should be expanded to give some responsibility to learners who feature in the programme as mere participants.
